# Transposable Elements and Stress in Vertebrates: An Overview

**DOI:** 10.3390/ijms22041970

**Published:** 2021-02-17

**Authors:** Anna Maria Pappalardo, Venera Ferrito, Maria Assunta Biscotti, Adriana Canapa, Teresa Capriglione

**Affiliations:** 1 Department of Biological, Geological and Environmental Sciences—Section of Animal Biology “M. La Greca”, University of Catania, Via Androne 81, 95124 Catania, Italy; vferrito@unict.it; 2 Department of Life and Environmental Sciences, Polytechnic University of Marche, Via Brecce Bianche, 60131 Ancona, Italy; m.a.biscotti@univpm.it (M.A.B.); a.canapa@univpm.it (A.C.); 3 Department of Biology, University of Naples “Federico II”, Via Cinthia 21—Ed7, 80126 Naples, Italy; teresa.capriglione@unina.it

**Keywords:** transposable elements, vertebrates, environmental and dietary factors

## Abstract

Since their identification as genomic regulatory elements, Transposable Elements (TEs) were considered, at first, molecular parasites and later as an important source of genetic diversity and regulatory innovations. In vertebrates in particular, TEs have been recognized as playing an important role in major evolutionary transitions and biodiversity. Moreover, in the last decade, a significant number of papers has been published highlighting a correlation between TE activity and exposition to environmental stresses and dietary factors. In this review we present an overview of the impact of TEs in vertebrate genomes, report the silencing mechanisms adopted by host genomes to regulate TE activity, and finally we explore the effects of environmental and dietary factor exposures on TE activity in mammals, which is the most studied group among vertebrates. The studies here reported evidence that several factors can induce changes in the epigenetic status of TEs and silencing mechanisms leading to their activation with consequent effects on the host genome. The study of TE can represent a future challenge for research for developing effective markers able to detect precocious epigenetic changes and prevent human diseases.

## 1. Introduction

In the 1940s, when the Ac/Ds (activator/dissociation) transposon system was identified as the genetic basis for maize kernel variegation, Barbara McClintock proposed transposable elements (TEs) as genomic regulatory elements [1,2]. Later, Britten and Davidson suggested that repeated DNA components may affect gene expression through binding sites recognized by regulatory factors [3]. However, for several decades TEs were considered as molecular parasites, whose activity contributed to genetic instability by creating insertional mutations in genes, promoting double-strand breaks and interfering with the epigenetic status of genomes. By the end of the 2000s researchers abandoned this idea and an increasing number of papers has highlighted that TEs can be a source of genetic diversity and regulatory innovation, either promoting the emergence of novel genes or providing new regulatory regions [4,5]. Indeed, it is now recognized that the variability in number and composition of the TE families present in the genomes together with their ability to move represents one of the major forces for genome evolution [6].

**Figure 1:**
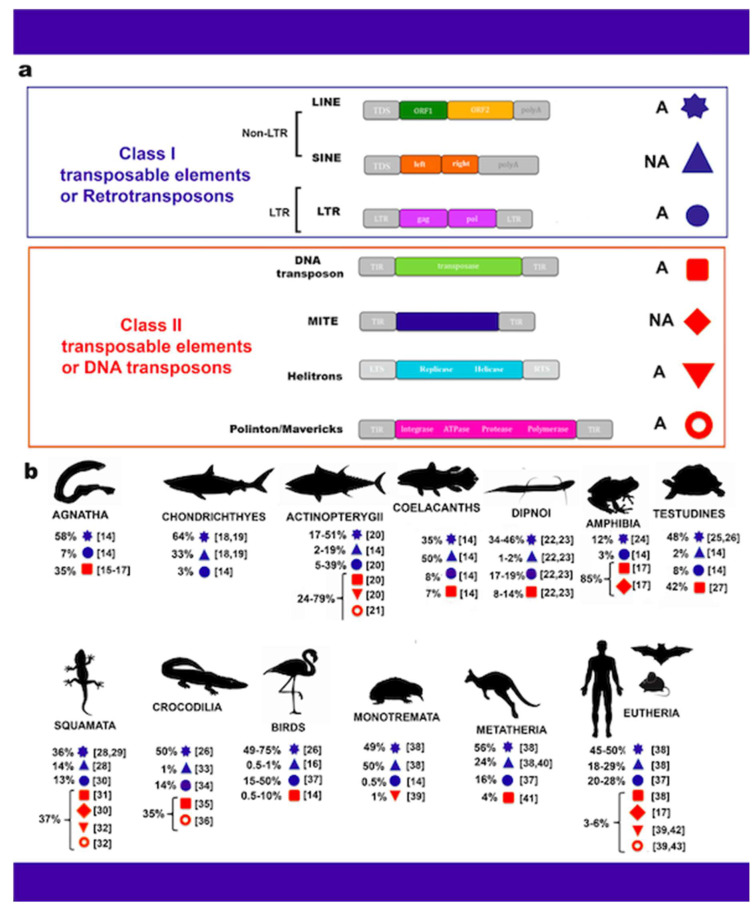
Classification (**a**) and distribution of transposable elements (TEs) in vertebrates (**b**) [14,15,16,17,18,19,20,21,22,23,24,25,26,27,28,29,30,31,32,33,34,35,36,37,38,39,40,41,42,43]. In panel (**a**), autonomous elements are indicated with capital letter A, while non-autonomous elements with capital letters NA. Symbols attributed to each transposable element type are reported on the right. ORF: open reading frame; polyA: polyadenylation site; *gag*: gene coding for the structural protein; *pol*: gene coding for reverse transcriptase, ribonuclease H, and integrase. TIR: terminal inverted repeat; TSD: target site duplication. LTS: left terminal sequence; RTS: right terminal sequence. Numbers in square brackets indicate references; the percentages indicate the relative amount of each TE type in different vertebrate genomes based on data from Chalopin et al. [15], Meyer et al. [22], and Wang et al. [23].

Sequencing of eukaryotic genomes has revealed that a consistent fraction of repetitive DNA is represented by TEs. The mechanism used by these genetic elements to move and insert into various host genomic positions is called transposition. TEs can be grouped into two major categories based on the way they relocate in the genome: class I retrotransposons and class II DNA transposons. Retrotransposons relocate indirectly via RNA intermediates using a copy and paste mechanism. These elements are divided into Long Terminal Repeat (LTR) retrotransposons and non-LTR retrotransposons on the basis of the presence or absence of flanking sequences [7,8] (Figure 1a). LTR retrotransposons are long, about 250–600 bp, and are essential for transcription and insertion in new host genome position. LTR retrotransposons present the whole set of genes useful for transposition and thus are able to autonomously replicate and move themselves. In particular, their structure, constituted by genes encoding for gag proteins, reverse transcriptase, protease, RNase H, and integrase, is similar to the that of retroviruses. Ty1/Copia (*Pseudoviridae*), Ty3-gypsy-like (*Metaviridae*), and Bell/Pao are the three main families of LTR retrotransposons spread among vertebrates. Non-LTR retrotransposons include Long Interspersed Nuclear Elements (LINEs) and Short Interspersed Nuclear Elements (SINEs). As LTR retrotransposons also, LINEs are autonomous elements while SINEs are non-autonomous retrotransposons that use the retrotranscription machinery of other TEs [9].

Class II DNA transposons are wholly DNA-based elements (found in both prokaryotes and eukaryotes) that can directly relocate autonomously from DNA to DNA, via a cut and paste mechanism. However, about a decade ago, analysis of a large amount of eukaryotic genome sequences led to the discovery of a new type of DNA transposons called Polintons (known also as Mavericks) and Helitrons [10,11,12,13]. Most of the Polintons encode homologs of major and minor icosahedral viruses, capsid proteins and several enzymes such as integrase, ATPase, protease, and polymerase. Helitrons encode for Rep/Helicase protein having endonuclease and helicase activity and are able to move by rolling-circle replication mechanism [10]. Differently from these DNA transposons, Miniature Inverted-repeat Transposable Elements (MITEs) are non-autonomous elements less than 800 bp long with terminal inverted repeats [6] (Figure 1a).

The various classes of TEs are differently represented in terms of both quantity and quality across all domains of life. For example, 45% of the repeated elements in the human genome is represented by retrotransposons [44] while plant genomes may hold a higher proportion of transposable element-derived DNA [45,46]. Indeed, more than 80% of the genomes of barley, wheat, and maize consist of DNA transposons [47]. All classes of TEs are present in the *Arabidopsis* genome, while *Saccaromyces cerevisiae* contains only members of the LTR retrotransposon family. A wide variety, present in limited numbers, of LTR and non-LTR retrotransposon families are present in the genome of *Drosophila*. Conversely, a very large number of few related retroelements from the IAP (Intracisternal-A-particle) (LTR), LINE1, and SINE B1 (non-LTR) retrotransposon families dominate the mouse genome [48]. The remarkable ability of TEs to colonize genomes was further confirmed by the recent discovery of TEs in giant virus DNA [49].

Vertebrates are the sister group of urochordates and their common ancestor diverged from cephalochordates about 500 Mya [50]. Vertebrates experienced innovations and adaptations that allowed the extraordinary evolutionary success of this group. Several bursts of TEs, belonging to different families, have affected the genome of vertebrates over this time [51,52] and the subsequent exaptation of new TE insertions may have conditioned genome evolution and provided raw material for transcription factor binding sites. Indeed, it is not negligible that an increasing amount of evidence supports the origin of transcription factor binding sites and non-coding RNAs from TEs [53,54,55]. In humans, about 20% of conserved regulatory sequences have been co-opted from TEs [56].

Epigenetic mechanisms such as DNA methylation and histone modifications, as well as small RNAs and sequence-specific repressors such as KRAB zinc-finger proteins, regulate the activity of TEs, generally silencing them. TE reactivation and retro-transposition have been reported in the development of human pathogenesis and environmental stressors seem to play a major role in determining these events. Therefore, TEs are more than junk and have a significant role in maintaining the genome structure and stability, but they are also involved in the onset of diseases and, in this context, an important open question is to what extent diet could affect TEs mediated gene expression. In light of these premises, in this review, we present an overview of the impact of TEs on vertebrate genomes, report the silencing mechanisms adopted by host genomes to regulate TE activity, and finally we explore the effects of environmental factor exposures on TE modulation in mammals which is the most studied group among vertebrates.

## 2. TEs in Vertebrate Genomes

Innovations in cells, tissues, organs, and structures have been responsible for the evolution of new species in vertebrates. Many of these modifications have been linked to TE activity and in particular to exaptation events that contribute to the creation of new genes and regulatory elements [57]. The comparison with cephalochordates, the living organisms closest to the chordate ancestor, has highlighted that vertebrate genomes have experienced a general decrease of TEs in terms of quantity and diversity [58]. However, not clear is the condition of organisms having large genome size such as lungfish and salamanders, for which genome assemblies are absent or limited and thus information on TEs at genome level are rather scarce. Overall the various classes of TEs are differently represented across vertebrate lineages and some TE superfamilies are widespread among vertebrate lineages, while others present a patchy distribution indicating that events of loss or gain occurred during evolution [14].

For Agnatha, genome assemblies are currently available for only three species: the hagfish *Eptatretus stouti* and the lampreys *Lethenteron camtschaticum* and *Petromyzon marinus*. TEs have been investigated only in *P. marinus* in which these elements constitute the 34.7% of the assembled sequences and are mainly dominated by LINE retroelements followed by DNA transposons [14,59,60]. Among Gnatosthomata, and also Chondrichthyes, data available are restricted to a single species, the elephant shark *Callorhinchus milii* [61], in which TE-derived DNA makes up 42% of the total sequenced genome with a prevalence of LINEs followed by SINEs. TE content is highly variable in fish, ranging from 5% to 56% of the genome (Figure 1b). Moreover, they present the highest TE diversity among vertebrates, a feature that is maintained also in organisms with small genomes as in Tetraodontiformes. The mobilome of fish is predominantly represented by DNA transposons and LINEs, whereas SINEs are the least abundant. However, in the non-teleost *Lepisosteus oculatus* TE content is enriched by retroelements [14]. Furthermore, a statistically significant correlation has been detected between TE content and the size of fish genomes [62]. In teleost, lineage-specific TEs such as Rex1, Rex3, and Rex6 elements have received special attention due to the role they have likely played in the evolution of teleost fish [63,64,65,66]. Recently, a novel database (FishTEDB) has been developed including 27 bony fishes, 1 cartilaginous fish, 1 lamprey, and 1 lancelet to provide a good basis for TE functional studies and promote transposable element research [67].

It is interesting that the condition of coelacanths shows some very active TE families despite their slow evolving genome [68,69]. The mobilome of these basal sarcopterygians, corresponding to about 20% of their genome, is dominated by SINE retroelements [14].

The huge size of the lungfish genome has long represented a challenge for assembly procedures. However, the recent genome sequencing of the Australian lungfish *Neoceratodus forsteri* and the West African lungfish *Protopterus annectens* allowed the gap in our knowledge about this taxonomic group to be filled. The analysis in *N. forsteri* revealed that about 90% of the assembly is made up of TEs [22] while 61.7% in *P. annectens* [23]. Both analyses showed that LINE retrotransposons represent the major amount in agreement with a previous report [70] and the elements are also highly active in *P. annectens* [62].

Similarly, Amphibia is a clade characterized by organisms with large genomes as the case of some caecilians with approximately 14 Gb up to extreme values in salamanders with about 120 Gb [71]. These differences have been attributed to various factors such as the presence of longer introns [72] and a low rate of DNA elimination [73] but also to the variable propensity towards TE accumulation.

Indeed, the two *Xenopus tropicalis* and *Nanorana parkeri*, having genomes of 1.5 Gb and 2.3 Gb respectively, differ in their TE content of about 25% for the Western frog and about 50% for the Tibetan frog [74]. This incongruence was related to the expansion of DNA transposons in *X. tropicalis*, in particular of Kolobok-T2 elements and of LTR retrotransposons in *N. parkeri*, in particular of Gypsy elements [74]. These retroelements have been found to be really abundant also in the highly repetitive genome of the strawberry poison frog *Oophaga pumilio* [75] and have been proposed also as responsible for the gigantism of salamanders [73]. Instead, the recent analysis performed on the caecilian *Ichthyophis bannanicus* genome evidenced an abundance of *Dictyostelium* intermediate repeat sequence (DIRS) and LINE/Jockey elements [76]. The genomes of non-bird reptiles examined to date showed a TE content ranging from 20% to 30% with a prevalence of LINE retroelements and DNA transposons [32,77,78,79]. Moreover, a decrease of TE superfamily richness was reported for this taxon indicating that a progressive reduction of TE diversity occurred during sarcopterygian evolution [14]. This aspect is extreme in the small genomes of birds which present low TE copy number and diversity dominated mainly by CR1 elements [80]. However, the recent study of Suh et al. [81] demonstrated that the flycatcher and the zebra finch lineages exhibit a high diversity of novel, lineage-specific retrovirus-like LTR retrotransposons.

The complete DNA sequencing of whole mammalian genomes has shown that at least 46% of human DNA, 31% of canine DNA, and 37% of mouse DNA are derived from TEs [82]. In most mammalian genomes a large proportion of TEs consists of LINE and SINE retrotransposons, more limited are LTR retrotransposons and minimal DNA transposon. Among LINE elements, LINE1 (L1) is the most active in the human genome as well as SINE elements [83]. The mammalian TE repertoire differs for the diversity of SINE retroelements that are order/family-specific and emerged independently multiple times [84]. Although it has been highlighted that human DNA transposons have accumulated mutations rendering them immobile, potentially active DNA transposons have been identified in the genome of bat species [38,82].

## 3. Regulation Mechanisms of TE Activity

**Figure 2:**
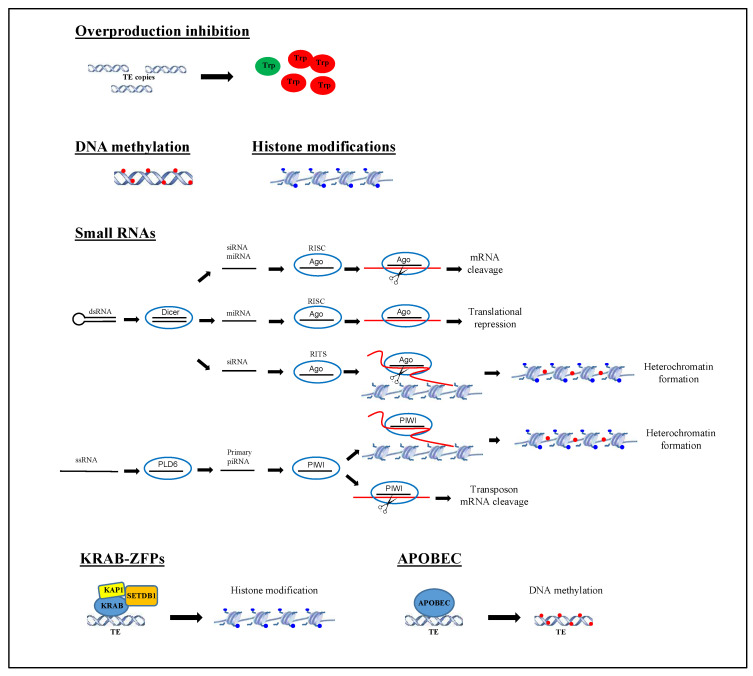
Transposable element silencing mechanisms. The main mechanisms involved in silencing transposable elements are represented. For details see text at paragraph 3. Active transposase is represented with green ovals; inactive transposases are represented with red ovals; modifications at DNA level are represented with small red circles; modifications at histone tails are represented with small blue circles; a red line indicates mRNA; a long red curved line indicates nascent RNA.

Changes of genome size and composition are related to TE mobilization. Although the trigger involved in TE induction is not completely understood, it is clear that the transposition process can cause deleterious effects in the host genome with consequent reduction of fitness and thus also of TE replication and propagation. Therefore, strategies to minimize the negative impact of TE mobilization have been developed. Some TEs have evolved self-regulatory mechanisms controlling their own copy numbers [85,86]. In this regard, a self-regulatory mechanism has been described for DNA transposons of the superfamily Tc1/mariner, the so-called overproduction inhibition (OPI). The increase of copy number leads to an increase of synthetized transposases that in turn determine a higher transposon activity. The OPI mechanism consists in the formation of inactive or less inactive transposase oligomers to decrease transposition [87]. On the other side, the controlling TE mechanisms from the host are: (i) DNA methylation, (ii) histone modifications, (iii) regulation by small RNAs, (iv) sequence-specific repressors such as the recently profiled Krüppel-associated box (KRAB) zinc-finger proteins [88,89,90], and (v) sequence editing by apolipoprotein B mRNA editing enzyme, catalytic polypeptide-like (APOBEC) (Figure 2).

DNA methylation is the most widely adopted mechanism for silencing TEs and consists of the addition of methyl groups to DNA. In particular, a DNA methyltransferase catalyzes the transfer of a methyl group from S-adenyl methionine to the fifth carbon of a cytosine residue to form 5-methylcytosine. This process is connected with the cytosine-guanine dinucleotide (CpG) sites. It is not surprising that about 90% of methylated CpG sites in the mammalian genome are found in repeated elements and above all in TEs such as SINEs and LINEs [91]. Besides this strategy, recent investigations have pointed out the key role of histone modifications as one of the most important epigenetic mechanisms in regulating TE activity [92]. In fact, changes at post-translational level of N-terminal histone tails such as acetylation, methylation, and phosphorylation can prevent the accessibility of DNA to regulatory factors and/or polymerase complexes by affecting inter-nucleosomal interactions, chromatin structure, and finally gene expression.

Another conserved mechanism of silencing of TEs includes the control by regulatory non-coding RNAs (ncRNAs), such as microRNAs (miRNAs), short interspersed RNAs (siRNAs), and PIWI-interacting RNAs (piRNAs). These ncRNAs differ for number of nucleotides, origin, and function. miRNAs and siRNAs have a length of about 21–25 nucleotides and are generated from double-stranded RNA precursors cleaved by Dicer. miRNAs and siRNAs become part of a cytoplasmatic RNA-induced silencing complex (RISC) including Argonaute subfamily proteins, and are able to bind target mRNAs and prevent translation [93]. siRNAs are part of the RNA-induced transcriptional silencing complex (RITS) containing an argonaute-family protein. The small RNA is used to target nascent RNAs still attached to RNA polymerase and DNA. The transcripts are cleaved and the involved DNA regions undergo modifications of chromatin as the methylation at lysine 9 of histone H3 and DNA methylation [94]. Mechanisms that restrict TE expression and mobilization are likely to be particularly important in germ cells and piRNAs represent the master regulators in vertebrates [95,96]. piRNAs have a size of about 24–31 nucleotides and are associated with PIWI subfamily proteins. The precursors of piRNAs are long single-stranded RNAs and for this reason they cannot be processed by Dicer. These precursors are cleaved by an endonuclease Phospholipase D Family Member 6 (PLD6) producing the 5′ end of primary piRNAs and then loaded into Piwi proteins before again being trimmed to generate the 3′ end of the mature piRNAs [97]. Moreover a ping-pong pathway involving PIWI proteins is responsible for the production of secondary piRNAs. Silencing of TEs can take place transcriptionally through histone and DNA methylation of TEs, as well as post-transcriptionally by targeted TE transcript degradation [57,98]. The Krüppel-associated box domain zinc finger proteins (KRAB-ZFPs) and the APOBEC enzymes represent vectors for these types of TE regulation. The former are a family of transcriptional regulators, that control TEs in higher vertebrates including humans acting during embryonic development [53]. They recognize TEs in a sequence-specific manner and recruit the co-factor KAP1, and the histone methyltransferase SETDB1 that is involved in the deposition of H3K9me3 modification. After this step, TEs are de novo methylated at DNA level and this epigenetic change persists in somatic and germ cells [99].

A wealth of data indicates that the evolutionary history of KRAB-ZFPs is strictly linked to that of TEs. Indeed, TEs can evade detection by host KRAB-ZNFs by acquiring mutations and consequently the host repressor mechanisms need to evolve rapidly in order to maintain genome integrity. Two models have been hypothesized to explain the relationship between KRAB-ZFPs and TEs. The first is consistent with an “arms race model” stating that the major role of KRAB-ZFPs is the recognition and transcriptional silencing of TEs as recently demonstrated by large scale ChIP-seq studies and loss-of-function experiments [87]. However, this model has been considered too simplistic to justify the selection of KRAB-ZFPs genes. On the other hand, the “domestication model” introduces the concept that TE sequences could be co-opted to serve cellular functions beneficial to the host organism [100]. For this reason, a KRAB-ZFP recognizing a TE could either silence it, modify its regulatory activity, or leave it intact [90]. The APOBEC enzymes, most of which are potent DNA cytidine deaminases, through DNA editing, convert cytosines residues to uracils (C-to-U) in a wide variety of parasitic elements, including many retroviruses [81]. This mechanism leads to an accumulation of mutations that render TEs inactive [101]. However, it has been demonstrated that in a few cases a preferential retention of edited elements bearing high mutation loads could give rise to a beneficial sequence that is positively selected and retained in active genomic regions [81].

## 4. How Environmental Stresses Modulate TE Activity

TE activation in response to environmental stress was first proposed by Barbara McClintock [2], who believed that controlling elements (TEs) permitted the genome to respond more flexibly to environmental shocks and stresses. The hypothesized mechanism was the association of transposition with heterochromatin and the subsequent alteration of gene expression through mobilization of heterochromatin domains [2].

In general, environmental stressors, natural or of anthropogenic nature, have been shown to alter epigenetic modifications such as DNA methylation and histone modifications, and the expression of small non-coding RNAs [102,103]. Failure of silencing mechanisms results in TE reactivation that causes genomic instability with potentially neutral, harmful, or beneficial effects on the host genome [104,105]. Stress-activated TEs can provide raw material from which to generate new genes, or disseminate regulatory elements to create stress-inducible genes and/or networks, or act directly on specific genes. The activation of TEs in response to stress conditions has been suggested to have an adaptive role since transposition generates a higher mutation rate that causes an increase in genetic variability on which natural selection can act to generate advantageous functions for species to survive stressful situations. In mammals, the proopiomelanocortin gene (Pomc) is a gene expressed in a group of neurons of the hypothalamus and is involved in the regulation of food intake and energy balance. This gene is regulated by two enhancers originated from independent exaptation events of two unrelated retrotransposons. It is conceivable that these events occurred in response to periods of climate change during mammal evolution conferring an adaptive advantage through the inhibition of foraging behavior in the presence of predators and escaping after injury [106].

The exposition to numerous environmental stresses, such as exposure to pollution, terrestrial and space radiation, temperature changes, endrocrine distruptors, and physiological and psychological stresses, determines most frequently the hypomethylation of L1 retroelements and ERVs and the hypermethylation of Alu elements with consequently cancer and disease development in mammals [107]. Alu retroelements are hypomethylated in the peripheral blood cells in the case of exposure to persistent organic pollutants (many of which are endocrine disruptors) [108] and in tibia in the case of exposure to lead [109].

Exposure to stressful conditions can cause not only changes in TE transcription but also in transposition rate as in the case of heavy metal exposure. Indeed, arsenic [110] and mercury [111] can cause mobilization of L1 retroelements.

In mammals, the L1 element is one of the most abundant retrotransposons whose activity is responsible for a variety of disorders, from hemophilia A [112], diabetes, and β-thalassemia [113,114] to cancer [115,116,117,118]. Under normal conditions, L1 is silenced by the longevity regulating protein Sirtuin 6 (SIRT6) that keeps its promoter in a heterochromatic state, but under genotoxic stressors or during aging this mechanism fails and L1 is transcribed contributing to the development of age-related diseases [119]. Dysregulation of transposable elements has also been related to a variety of nervous system disorders [120,121,122] and physiological and psychological stresses have been proposed as a cause of TE activation [123]. The dynamics of the N(6)-methyladenine (6mA), a modified DNA adenine found in mammalian cells, have been examined in the mouse brain in response to environmental stress. A correlation between the gain of 6mA on intergenic regions and the downregulation of 90.4% of LINE retrotransposon expression has been found upon stress [124]. The regulatory effect of environmental stress on retrotransposon expression in the brain has been demonstrated also by Hunter et al. [125]. According to their findings, the initial activation of TEs can be followed by the repression of specific TEs, such as intracisternal A particle endogenous retrovirus elements and B2 short interspersed elements, to limit genomic instability. In the rat hippocampus, acute stress increases levels of the repressive histone H3 lysine 9 trimethylation (H3K9me3) at transposable element loci, correlated also with an upregulation of Suv39h2 gene encoding for the enzyme involved in the deposition of this epigenetic mark. The specificity of this TE regulatory mechanism in the hippocampus has been related to the presence of pyramidal cells involved in long-term memory whose instability would reduce the ability to maintain their functions [126].

Response to heat-shock mediated by TEs is one of the best stress statuses studied in vertebrates. In humans in the presence of heat stress the expression of some genes decreases while that of Alu elements increases. Indeed, it has been demonstrated that mRNAs produced from SINEs act in trans repressing the transcription by directly binding RNA polymerase II forming stable complexes at promoters [127]. In mice, the chaperone heat-shock protein HSP90 forms a complex with KAP1 protein to repress the regulatory influence of endogenous retroviruses on neighboring genes. Under stress conditions, this function is compromised and an upregulation of nearby genes by ERVs is detected [128]. This kind of stress can cause also both up- and downregulation of gene expression after TE activation. Indeed, the mRNA produced from B2 SINE retrotransposon normally binds stress-responsive genes, decelerating the progression of RNA polymerase II and thus reducing their expression. However, stress induces the recruitment of EZH2 protein that in turns cleaves B2 RNAs causing an increased expression of stress genes. At the same time, B2 RNA molecules bind other genes downregulating them [129].

In humans in vitro, in vivo, and epidemiological studies evidence that TEs are sensitive endpoints for detection of the effects caused by environmental stresses suggesting their use as biomarkers and/or targets for therapeutic treatment and prevention of diseases [107].

Overall, a specificity seems to exist between TEs and stress condition [104] but the behavior of stress-induced TEs seems to depend on the type of stress and TE [130]. However, several open questions remain to be elucidated concerning the relationship between TEs and stress as to why some TEs are upregulated and others downregulated or why some TE families are more prone to be activated than others [130].

## 5. How Nutrition Modulates TE Activity

Since the pioneering investigations of Wolf et al. [131], Morgan et al. [132], and Waterland and Jirtle [133], environmental epigenomics has become a prolific field of research. These authors showed that maternal dietary supplementation with methyl donors such as folic acid, choline, and betaine caused a shift in the coat color of agouti mice offspring from yellow to brown. Furthermore, they identified the DNA methylation of a transposable element upstream of the *agouti* gene, as a molecular basis for the observed color shift. Since then, a series of research has led to the rise of nutritional epigenomics as a sub-discipline of environmental epigenomics, focused on the role of dietary factors (micro-nutrients, macro-nutrients, and non-nutrient dietary components) on epigenetic modifications and disease risk [134]. Additional evidence for the diet-dependent epigenetic repression of TEs has been found.

For example, the study of Dolinoy et al. [135] demonstrated the epigenetic effects of prenatal exposure to bisphenol A (BPA) in mice leading to yellow fur, obesity, diabetes, and tumorigenesis. In particular, the authors highlighted that maternal BPA exposure reduces DNA methylation at nine CpG sites located immediately upstream in the cryptic promoter region of the *A^vy^* IAP retrotransposon. More importantly, the authors discovered that maternal nutritional supplementation with methyl donors or genistein counteracts BPA-induced hypomethylation restoring the coat color distribution in BPA-exposed offspring toward the methylated pseudoagouti phenotype. The covalent binding of the vitamin biotin to lysine-12 in histone H4 (H4K12bio), mediated by holocarboxylase synthetase (HCS), has been demonstrated to be responsible for the repression of LTR retrotransposons in human and mouse cell lines [136]. The authors showed that repression of LTR retrotransposons depends on crosstalk between H4K12bio and methylation marks and proposed a model in which three nutrient-dependent repression marks (cytosine methylation, H4K12bio, and H3K9me2) synergize in the repression of LTR retrotransposons. Additionally, Kuroishi et al. [137] clearly demonstrated that biotinylation is a rare but natural histone modification in humans. The ability of natural stilbenoids deriving from resveratrol to inhibit the HIV-1 integrase, a protein involved in the integration mechanisms of retroviruses, and the eukaryote MOS-1 transposase, a protein responsible for the mobility of the mariner group transposable elements, have been demonstrated in vitro and in vivo by Pflieger et al. [138]. By assessing the efficacy of two resveratrol dimers and fourteen stilbenoids against the two enzymatic models, the authors found a different activity of the molecules, some of which were active against both proteins while others were specific for one of the two models, suggesting that specific intermediate nucleocomplexes of the reactions could be targeted by the compounds. The results obtained by Agodi et al. [139] demonstrated that a dietary pattern characterized by low fruit consumption and folate deficiency, not adherent to the Mediterranean dietary pattern, was associated with LINE-1 hypomethylation and with cancer risk in 177 healthy women. The authors suggest that leukocyte LINE-1 methylation may serve as a biomarker for dietary interventions designed to reduce the risk of cancerous and precancerous conditions. Furthermore, Barchitta et al. [140] examined a sample of 299 healthy women and demonstrated for the first time the inverse association between adherence to the Mediterranean diet and exposure to particulate matter having an effective aerodynamic diameter smaller than 10 μm (PM10) with LINE-1 methylation. More specifically, they showed that the monthly PM10 exposure level was significantly and inversely associated with LINE-1 methylation which, on the contrary, was significantly and positively associated with adherence to the Mediterranean diet measured through the Mediterranean Diet Score. Interestingly, studies by Green et al. [141] additionally demonstrated that lifespan-extending diets in mice largely repressed the expression of miRNAs, lncRNAs, and TEs. In particular, the authors characterized lifespan-related liver transcriptome changes mediated by different dietary intervention regimens. They found that coding genes, repeat elements and miRNAs were regulated by dietary interventions and were highly correlated with lifespan and aging. The authors suggested that a crosstalk exists among miRNAs, chromatin remodelers, and TEs: they identified specific miRNAs (miR-34a, miR-107, and miR-212-3p) targeting Chd1, a chromatin remodeler gene, able to both activate transcription and repress it. On one hand Chd1 promotes the expression of mRNA positively related with lifespan, on the other it represses transposable elements.

## 6. Conclusions

**Figure 3:**
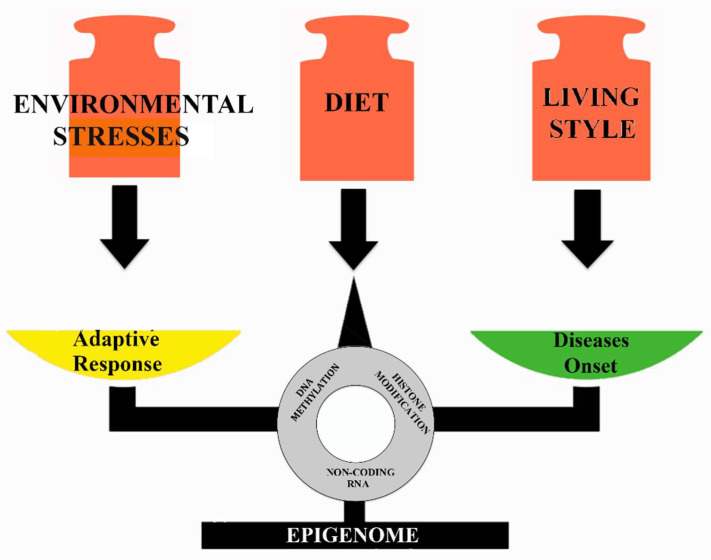
Environmental and nutritional epigenetic effects on the health status.

Transposable elements make genomes dynamic and are responsible for their evolution. It is known that the quantitative impact of total TEs as well as of specific TE types varies in different lineages. Our comparative analysis of TE accumulation in vertebrate genomes revealed that information is mainly focused on mammals, while for many other groups information on TEs is rather scarce or even absent due to the lack of available genome assemblies. Moreover, in the last decade, a significant number of papers have been published highlighting a correlation between TE activity and exposition to environmental stresses and dietary factors. In general, they cause changes in DNA methylation, histone modifications, and in the expression of small non-coding RNAs. These modifications determine the failure of silencing mechanisms with consequent reactivation of TEs. This activity can have an adaptive role generating a higher mutation rate that causes an increase in genetic variability on which natural selection can act to generate advantageous functions for species to survive stressful situations. On the contrary, transposition can have deleterious effects in the host genome and cause the onset of diseases. Certainly, many questions remain to be addressed, such as to unveil the TE diversity and function in a wider range of species than that known today, to detect the wide variety of propagation mechanisms of TEs within genomes, and finally to understand to what extent diet could effect TEs mediated gene expression. The deep knowledge of these aspects will allow the interplay between TE intrinsic characteristics, host biology, and response to the environmental factors (Figure 3) to be correctly evaluated.
